# Design and Sampling Plan Optimization for RT-qPCR Experiments in Plants: A Case Study in Blueberry

**DOI:** 10.3389/fpls.2016.00271

**Published:** 2016-03-07

**Authors:** Jose V. Die, Belen Roman, Fernando Flores, Lisa J. Rowland

**Affiliations:** ^1^U.S. Department of Agriculture, Agricultural Research ServiceBeltsville, MD, USA; ^2^Area Mejora y Biotecnologia, IFAPA Centro Alameda del ObispoCordoba, Spain; ^3^Departamento Ciencias Agroforestales, Universidad de HuelvaHuelva, Spain

**Keywords:** blueberry, confounding variation, qPCR, replicates, RT variability

## Abstract

The qPCR assay has become a routine technology in plant biotechnology and agricultural research. It is unlikely to be technically improved, but there are still challenges which center around minimizing the variability in results and transparency when reporting technical data in support of the conclusions of a study. There are a number of aspects of the pre- and post-assay workflow that contribute to variability of results. Here, through the study of the introduction of error in qPCR measurements at different stages of the workflow, we describe the most important causes of technical variability in a case study using blueberry. In this study, we found that the stage for which increasing the number of replicates would be the most beneficial depends on the tissue used. For example, we would recommend the use of more RT replicates when working with leaf tissue, while the use of more sampling (RNA extraction) replicates would be recommended when working with stems or fruits to obtain the most optimal results. The use of more qPCR replicates provides the least benefit as it is the most reproducible step. By knowing the distribution of error over an entire experiment and the costs at each step, we have developed a script to identify the optimal sampling plan within the limits of a given budget. These findings should help plant scientists improve the design of qPCR experiments and refine their laboratory practices in order to conduct qPCR assays in a more reliable-manner to produce more consistent and reproducible data.

## Introduction

Since Russ Higuchi published his paper describing the use of real-time quantitative PCR (qPCR) (Higuchi et al., 1993), it has become one of the most popular techniques in modern molecular biology. Once adopted by the research community, its use increased dramatically with a growth curve resembling the sigmoidal amplification plots that are obtained during the qPCR experiment itself (VanGuilder et al., 2008). Although 2014 was the first year in which there was a reduction in the number of publications using qPCR (Huggett et al., 2015), the technology is, without doubt, the most widely used technique for the detection and quantification of nucleic acids, and in particular, it is the most common method for determining gene expression levels.

The skeptical nature of science is evident in the assumption of the null hypothesis as a starting point, e.g., there is no difference in the expression of a gene between two biological samples. A reverse transcription-qPCR (RT-qPCR) experiment is designed to test the null hypothesis. If, according to experimental data, the null hypothesis is rejected, the alternative hypothesis must be concluded. To obtain consistent, reproducible, and biologically relevant qPCR measurements, researchers must complete a number of complex technical steps, all of them influencing the accuracy and precision of results (Udvardi et al., 2008; Regier and Frey, 2010; Graeber et al., 2011; De Keyser et al., 2013; Remans et al., 2014). Two of the main issues that have caused debate in the plant research community are concerns over RNA quality assessment and data normalization based on multiple, assay-validated reference genes (Gutierrez et al., 2008a,b; Guénin et al., 2009; Die et al., 2011; Die and Román, 2012). However, it is astonishing how few publications have explored the reliability and reproducibility of results on the basis of the experimental design itself (Rieu and Powers, 2009).

A typical real-time qPCR experiment is comprised of several sample-preprocessing steps that are necessary before the actual amplification of the cDNA by the qPCR instrument. Typical steps include: (1) the sampling of material and the extraction of the RNA; (2) the reverse transcription to convert it into cDNA; and, finally, (3) the amplification of the cDNA by qPCR. All of these steps are susceptible to the introduction of error (Bustin and Nolan, 2004; Bustin et al., 2013), and combined they represent the technical noise that contributes to the total variance of the obtained measurement. An optimal experimental design aims to minimize sources of confounding variability. This objective can be achieved through effective, informed designs and sampling plans that employ replicates where they are expected to have the greatest benefit (Kitchen et al., 2010).

During the evolution of the qPCR technology, there was early recognition of technical limitations, particularly that reverse transcriptases (RTases) differed in their ability to transcribe and the reverse transcription (RT) step itself was extremely variable representing an important impediment to reliable data interpretation (Bustin and Dorudi, 1998). In 2004, Ståhlberg and collaborators provided the first empirical evidence for high variability being an inherent property of the reverse transcription step (Ståhlberg et al., 2004a,b). The authors concluded that assays should be run in (at least) duplicates starting with the RT reaction. In a follow-up publication, a nested experimental design was used to estimate the errors of sample extraction, RT, and qPCR that are introduced into measurements in solid tissue, blood, cell culture, and single cells from animals (Tichopad et al., 2009). The study provided support for the use of duplicates but, even more relevant here, it pointed out that the use of replicates relies on both, the noise contributed by that particular step and the noise contributed by subsequent steps. Therefore, in a frame of stage-of-sampling-dependent confounding variation, an optimal experimental design should be planned by using stage-of-sampling-dependent replicates.

This observation might be one of the most overlooked recommendations for qPCR analysis improvement. It has been argued that these papers place question marks around many of the results reported in the biomedical literature (Bustin, 2014), not to mention the plant science literature, which frequently reports modest differences in the expression levels of mRNA from an experimental design of 1 sample × 1 RNA × 1 RT × 2-3 qPCRs. The rationale behind the common practice of using technical replication at the qPCR level only is not clear. Also intriguing is the practice of performing statistical analyses based solely on replicates of the qPCR reaction.

In our own laboratory, much of our research is focused on measuring gene expression in the woody perennial fruit crop, blueberry, in response to abiotic stress and during development with the goal to identify genes of horticultural value. Traditional breeding efforts in blueberry have focused on the development of cultivars with broad climatic adaptation, season extension, disease and pest resistance, mechanical handling tolerance, and high fruit quality (Galleta and Ballington, 1996). If genes controlling these traits could be identified, marker-assisted selection could be used to facilitate blueberry breeding. Marker-assisted breeding would be particularly useful in blueberry, like some other woody perennials, because of long generation times, high heterozygosity, self-infertility, inbreeding depression, and polyploidy of commercial types (Rowland et al., 2014). Recently, there have been efforts by several laboratories, including our own, to sequence the transcriptome of blueberry (Dhanaraj et al., 2004, 2007; Rowland et al., 2012; Zifkin et al., 2012; Gupta et al., 2015). In the process, these efforts have generated large collections of both Sanger- and next generation ESTs. These are valuable resources for identifying genes that are potentially differentially expressed in flower buds, leaves, stems and fruits and may play important roles in cold acclimation, chilling unit accumulation, and fruit development in blueberry and related species (Rowland et al., 2012).

In this paper, by estimating the components of confounding biological variation or technical noise throughout processing of plant tissue samples at different stages of qPCR, we aimed to develop an optimal qPCR experimental design for use in our own blueberry gene expression studies, and by extension, by plant scientists in general, These stages represent inter-RNA sample, inter-RT, and inter-qPCR. Estimating the components of technical noise at each stage of sampling will allow us to further determine optimal experimental designs and sampling plans as well as maximize the statistical resolution of the assays. We performed several qPCR assays arranged in a hierarchical structure for different biological materials, for several genes and for several genotypes.

## Materials and methods

### Plant material and experimental design

Several fruits, leaves and stems were collected at the same time (August 15th, 2013) from one single plant of the rabbiteye blueberry (*Vaccinium virgatum*) cultivars “Tifblue” and “Premier,” both grown at the USDA/ARS, Beltsville Agricultural Research Center, Beltsville, MD. All tissues were frozen in liquid nitrogen immediately after harvest and stored at −80°C. The hierarchical experimental design was as follows: from each genotype three tissue samples (fruits, leaves and stems) were collected. Each tissue was split into 4 RNA extractions, then each RNA sample into 4 RT reactions and finally each RT reaction into 3 qPCR replicates. This design allowed us to investigate: (1) qPCR variability comparing the Cq ranges from 3 replicates; (2) RT variability by comparing the Cq ranges from 4 RT reactions; (3) tissue variability comparing the Cq ranges from 4 RNA samples. The nested design 1 plant × 3 tissues × 4 RNAs × 4 cDNAs × 3 PCRs produced a total of 144 Cq values per plant and qPCR assay.

### RNA isolation and quality controls

For total RNA isolation, each tissue sample made of several leaves, stems and fruits, respectively, was ground in liquid nitrogen and incubated at 65°C in a pre-warmed CTAB extraction buffer. Two or three chloroform:IAA (24:1) extractions were performed followed by overnight precipitation with LiCl (Chang et al., 1993). RNA pellets were resuspended in DEPC-treated water, precipitated again with ethanol and NaOAc, washed, and finally resuspended in 1 ml DEPC-treated water. RNA concentration was determined by measuring the optical density at 260 nm using a NanoDrop ND-1000 spectrophotometer (Nanodrop Technologies, USA). RNA quality was assessed by combining information from several control steps. First, purity was inferred from the absorption ratios using the NanoDrop. Only the RNA samples with *A*_260_/*A*_280_ ratio between 1.86 and 1.95 (leaves), 1.82 and 1.98 (stems), 1.71 and 1.84 (fruits) and *A*_260_/*A*_230_ greater than 2.0 (leaves and stems) or greater than 1.67 (fruits) were used in the analysis. Then, RNA samples were visualized on 1% agarose gels stained with ethidium bromide. Finally, we amplified segments of the 5′ and 3′ regions of an ubiquitin carboxyl-terminal hydrolase gene across the cDNA samples by qPCR, as described below.

### cDNA synthesis and quality controls

RNA extracts were treated with TURBO™ DNase I (Life Technologies, USA), prior to cDNA synthesis. The extracted RNA was split into 4 reverse transcription reactions. Two micrograms (leaves and stems) and ~1 microgram (fruits) of DNase I-treated total RNA were used for the synthesis of cDNA. Complementary DNAs was synthesized by priming with oligo-dT_12–18_ (Life Technologies, USA), using SuperScriptIII reverse transcriptase (Life Technologies, USA) following the instructions of the provider. The cDNAs were diluted to a final volume of 50 μl. Test for presence of genomic DNA (gDNA) contamination and qualitative assessment of the reverse transcriptase reaction and the RNA integrity were performed as have been described elsewhere (Die and Rowland, 2014). Briefly, we used a primer pair designed from two different exons of an alcohol dehydrogenase-like blueberry sequence that amplifies a product of 1140 bp using gDNA as template or 528 bp using cDNA as template. For assessment of the intactness of mRNA and the efficiency of cDNA synthesis we used a 3′:5′ amplification ratio assessment by measuring the integrity of an ubiquitin carboxyl-terminal hydrolase blueberry sequence (*UBP14*). The 3′:5′ amplification ratio of the *UBP14* cDNA fragments was calculated using the comparative C_q_ method. All ratios were inside the range of 1.02–3.96 (1.76 ± 0.67; mean ± SD). Only if ratios were >4.5-fold would RNA quality be deemed inadequate (Die et al., 2011). Therefore, the cDNAs were judged to be suitable for qPCR analysis.

### Primer design

Primer sequences were designed to amplify genes that may play important roles in different physiological functions that are the focus of our research, such as dormancy, cold acclimation and fruit quality in blueberry (B3 domain-containing TF VRN1-like, *VRN*; 3-ketoacyl-COA thiolase, *KAT*; flavonoid 3′,5′-hydroxylase, *F3*′*5*′*H*). The description of the sequences is shown in Supplementary Table 1. All PCR primers were tested for specificity using NCBI's BLAST software (Altschul et al., 1990). Primers were designed using the following criteria: Tm of 60 ± 2°C and PCR amplicon lengths of 65–100 bp, yielding primer sequences with lengths of 20–23 nucleotides and GC contents of 43–55% (Supplementary Table 2). For predicting the secondary structure of the amplicons, we used MFOLD version 3.4 software with default settings of minimal free energy, 50 mM Na^+^, 3 mM Mg^2+^, and an annealing temperature of 60°C (Zuker, 2003). We chose primers that would yield amplicons with minimal secondary structures and melting temperatures that would not hamper annealing (Supplementary Figure 1). Designed primers were synthesized by Integrated DNA Technologies (Coralville, IA, USA).

### Real-time qPCR

PCR reactions were carried out in an IQ5 (Bio-Rad, Hercules, CA, USA) thermal cycler using iQ™ SYBR® Green Supermix (Bio-Rad, Hercules, CA, USA) to monitor dsDNA synthesis. Reactions contained 1 μl of the diluted cDNA as a template and 0.150 μM of each primer in a total volume reaction of 20 μl. Master mix was prepared and dispensed into individual wells using electronic Eppendorf Xplorer® multipipettes (Eppendorf AG, Germany). The following standard thermal profile was used for all PCRs: polymerase activation (95°C for 3 min), amplification and quantification cycles repeated 40 times (95°C for 30 s, 60°C for 1 min). The specificity of the primer pairs was checked by melting-curve analysis performed by the PCR machine after 40 amplification cycles (60–95°C) and is shown in Supplementary Figure 2. Fluorescence was analyzed using iQ5 2.1 standard optical system analysis software v2.1 (Bio-Rad). All amplification plots were analyzed using a base line threshold of 30 relative fluorescence units (RFU) to obtain C_q_ (quantification cycle) values for each gene-cDNA combination. Supplementary Table 2 shows the overall mean real-time PCR amplification efficiency of each primer pair (E) estimated from the data obtained from the exponential phase of each individual amplification plot and the equation (1 + E) = 10^slope^ using LinReg software and the criteria of including three-five fluorescent data points with *R*^2^ ≥ 0.998 to define a linear regression line (Ramakers et al., 2003).

### Statistical design

The nested design or hierarchical structure used in this work have been previously defined by Tichopad et al. (2009). Briefly, the variance analysis was carried out with the PROC NESTED program in SAS software (version 9.1 for Microsoft Windows) with the linear model defined in Equation (1):

(1) $$\begin{array}{r} Cq_{ijkl}=\mu +a_{i}+b_{j\left(i\right)}+c_{k\left(ij\right)} \end{array}$$

where *Cq*_*ijkl*_ is the individual qPCR record that incorporates the total mean of the group (μ); the random effects of the *ith* sample (RNA, *a*_*i*_); the random effect of the *j*th RT reaction from sample *i* (RNA, *b*_*j*(*i*)_*)*; the random effect of the *k*th PCR reaction from the cDNA of the *j*th RT reaction transcribed from sample *i* (qPCR, *c*_*k*(*ij*)_). We applied the linear model of all hierarchical sampling processing effects within a single treatment group, or in our case, within a single tissue.

The total variance is given by Equation (2) and is denoted as $\sigma_{Cq}^{2}$.

(2) $$\begin{array}{r} \sigma_{cq}^{2}=\sigma_{i}^{2}+\sigma_{j}^{2}+\sigma_{k}^{2} \end{array}$$

where $\sigma_{i}^{2}$, $\sigma_{j}^{2}$, $\sigma_{k}^{2}$, are the variance contributions of the processing steps (sample, RT and qPCR levels, respectively).

The SD of a mean was calculated to analyze the noise reduction provided by the use of replicates at the level of the qPCR. The SD of a mean is the SE, which for an isolated processing step is:

(3) $$\begin{array}{r} SE=\sigma ∕\sqrt{N} \end{array}$$

The total expected variation that defines the optimal sampling plan can be calculated as follows:

(4) $$\begin{array}{r} \sigma_{cqg}^{2}=\sigma_{i}^{2}∕n_{i}+\sigma_{j}^{2}∕n_{i}n_{j}+\sigma_{k}^{2}∕n_{i}n_{j}n_{k} \end{array}$$

where $\sigma_{Cqg}^{2}$ is the total expected variance within a treatment group g (or variance of the mean Cq), and $\sigma_{i}^{2}$, $\sigma_{j}^{2}$,$\sigma_{k}^{2}$, are variance contributions of the processing steps: sample, RT and qPCR levels, respectively estimated from the pilot data. Additionally, *n*_*i*_ is the number of replicate samplings (RNA extractions) for each sample, n_*j*_ is the number of replicate RTs from each RNA sampling, and *n*_*k*_ is the number of replicate qPCRs from each RT.

### Code availability

R markdown and R code files used in order to identify the optimal sampling plan are available in a git-based, publicly accessible repository (https://github.com/jdieramon/BlueberryProject). We will continue to update and modify the code repository to meet the needs of users. However, older versions of the code can be retrieved using the command line-based git program which is well documented through numerous courses, tutorials and books available at many sites. The code is distributed under the open source MIT License.

## Results

We collected leaf, stem and fruit tissues from each of the two genotypes “Tifblue” and “Premier,” and performed 4 RNA extractions per tissue sample. Each extract was used as template for 4 RTs, each of which was run in 3 qPCR reactions. This design was analyzed for three genes: *VRN, KAT* and *F3*′*5*′*H* in singleplex format. Hence, we used a nested design (1 subject × 4 samples × 4 RTs × 3 qPCRs) that yielded 48 Cq values for each gene, tissue, and plant that sums up to a total of 864 qPCR reactions.

### Leaf tissue

**Figure 1:**
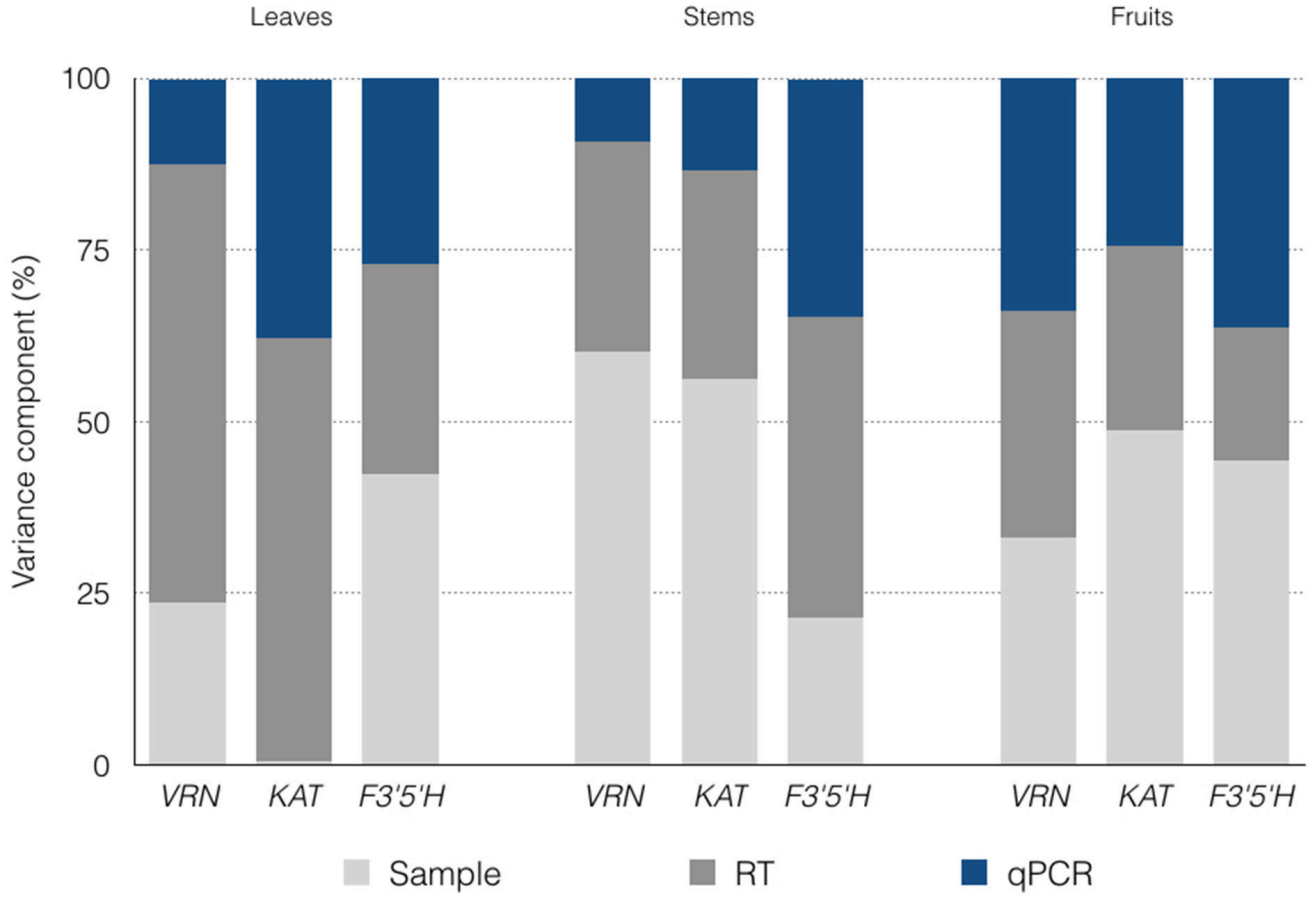
**Estimated confounding variation contributed by the sampling-processing steps**. The contributions to the overall noise are expressed as percentages.

Estimated SDs (σ) for the various processing levels are shown in Table 1. Also shown is the cumulative variation, which is expressed as the SD of measured Cq values $\left(\sigma_{\text{Cq}}^{\text{leaves}}\right)$ obtained from different plants. A total of 272 Cq values (16 missing) were measured for the three genes. *VRN* and *KAT* had Cq values < 26 cycles (*VRN* mean of 25.19 cycles, *KAT* mean of 22.78 cycles) whereas *F3*′*5*′*H* had lower expression with Cq mean of 30.82 cycles. The largest SD was estimated for the RT step, with $\left(\sigma_{\text{RT}}^{\text{leaves}}\right)$ values ranging from 0.28 to 0.73 cycles and mean value of 0.52 cycles. This value corresponds to a ~1.5-fold variation in RT yield. The qPCR showed the highest reproducibility, with $\sigma_{\text{qPCR}}^{\text{leaves}}$ values of 0.18–0.49 cycles (mean, 0.33 cycles). Mean $\sigma_{\text{Sampling}}^{\text{leaves}}$ values were 0.36 cycles but $\sigma_{\text{Sampling}}^{\text{leaves}}$ for *KAT* was negligible compared with the contribution of the subsequent steps. Expressed as variance contributions, the RT step accounted for ~50% of the total variation for the three genes (Figure 1).

**Table 1:** **SD estimates for sampling-processing steps and total noise (σCq)**.

	**Leaves**			**Stems**			**Fruits**		
	***VRN***	***KAT***	***F3′5′H***	***VRN***	***KAT***	**F3′5′H**	***VRN***	***KAT***	***F3′5′H***
Mean Cq	25.19	22.78	30.82	23.69	22.29	30.76	25.12	20.77	18.29
**SDs**									
**Processing noise**									
Sampling	0.45	0.02	0.64	0.87	0.53	0.19	0.41	0.42	0.43
RT	0.73	0.28	0.53	0.59	0.32	0.50	0.34	0.31	0.29
qPCR	0.32	0.18	0.49	0.35	0.21	0.44	0.30	0.27	0.35
Total noise	0.91	0.55	0.97	1.15	0.67	0.74	0.65	0.60	0.64

### Stem tissue

In total, 276 Cq values (12 missing) were measured with the nested design for the three genes. *VRN* and *KAT* showed similar range of quantification (mean Cq values of 23.69 cycles and 22.29 cycles, respectively) while *F3*′*5*′*H* showed lower expression with Cq mean of 30.76 cycles. This range (22.25–30.76) indicates that stem tissues showed the highest difference in expression levels between the three genes in our analysis (~355-fold). The estimated SDs were 0.19–0.87 cycles for $\sigma_{\text{Sampling}}^{\text{stems}}$ (mean, 0.53 cycles), 0.32–0.59 cycles for $\sigma_{\text{RT}}^{\text{stems}}$ (mean, 0.47 cycles) and 0.21–0.44 cycles (mean, 0.33 cycles) for the qPCR step, which showed the highest reproducibility (Table 1). Expressed as noise contributions, the sampling accounted for ~46% of the variation on average for the three genes and ~60% for *VRN* and *KAT* (Figure 1).

### Fruit tissue

We obtained a total of 284 Cq readings (4 missing) with the nested design for the three genes analyzed. Mean Cq values for the three genes were in the range 18–26 cycles. F3′5′H gene showed in fruits the highest expression level out of the three genes and the highest expression for every pair gene-tissue combination in the experiment. The largest estimate of SDs was for the sampling step, with ($\sigma_{\text{Sampling}}^{\text{fruits}}$) very similar values of 0.41–0.43 cycles (mean, 0.42 cycles); the highest reproducibility was observed in the RT step for gene F3′5′H (0.29 cycles) and the qPCR step for the other two genes with values of 0.27–0.30 cycles (Table 1). Our inspection of the variance contributions showed that sampling step dominated the overall error with a contribution of ($\sigma_{\text{Sampling}}^{\text{fruits}}$) ~42% (Figure 1).

## Discussion

### Modeling the technical noise

The larger the treatment effect, the easier it becomes to distinguish between the signal and the noise. The ability to resolve any differential expression between treatment groups diminishes due to the variance of available measurements, which includes the biological variation due to treatment (signal) and the confounding biological variation (technical or experimental noise). Although biological replicates are always necessary and provide confidence by increasing the number of observations of the given subpopulation, the use of technical replicates are effective in increasing precision. Therefore, biological replicates are required for making biological conclusions, whereas technical replicates are necessary for determining the technical validity of a method (Anon, 2013). In the “Minimum Information for the Publication of Quantitative PCR” guidelines (Bustin et al., 2009), the number of technical replicates and the workflow stage where they are performed is labeled as “essential information.” That means that that information should be submitted with the manuscript being available to editors, reviewers and readers.

Here, we have focused on the components of confounding biological variation, through the study of the introduction of error in qPCR measurements at different stages of the workflow. In a given qPCR experiment, it is assumed that the introduction of technical noise at each of the sampling levels is independent. However, the observed variances are not and the variation introduced at a given level propagates additively throughout subsequent levels, that is, the effect on the overall noise of the assay is additive. An experiment performed with a hierarchical structure, where clusters of replicates represent the integrated effects of the upstream processes, reflects that additive noise and that experiment may be used for modeling and estimating the components of noise directly from qPCR measurements (Tichopad et al., 2009).

In our objective to develop an optimal qPCR experimental design, we aimed to assess the contribution of individual processing steps to the overall noise, with the expectation that that knowledge will allow us to minimize the total expected variance through the use of technical replicates when they are expected to have the greatest benefit.

### Estimating the step that dominates the contribution of the variation

The total variance is given by Equation (2) and is denoted as $\sigma_{\text{Cq}}^{2}$. The corresponding SD is σ_Cq_. For the samples studied, our estimates are 0.85 cycles for $\sigma_{\text{Cq}}^{\text{stems}}$, 0.81 cycles for $\sigma_{\text{Cq}}^{\text{leaves}}$, and 0.63 cycles for $\sigma_{\text{Cq}}^{\text{fruits}}$. Therefore, the greatest variation is between stem tissues and this value may limit our ability to reliably detect small changes in gene expression, such as those performed by transcription factors. For example, reporting a variation of 2-fold (i.e., ~2^0.85^) for a given gene in stems might be suspicious because that level is well within the range of the expected confounding variation.

Once the overall confounding variation has been assessed for each tissue, we can analyze the variation contributed by the processing steps. For any tissue analyzed, there was one step that dominated the overall error. For leaves, the RT step accounted for ~26–72% of the total variation (on average 52% of the total variation), whereas for stems and fruits, the sampling step dominated the overall error with an average contribution of ~46 and 42% of the total variation, respectively. However, the error distribution may also have been gene dependent. For example, for the F3′5′H gene, which was expressed at the lowest level in stems, the $\sigma_{\text{Sampling}}^{\text{stems}}$ was only 0.19 cycles representing 21.5% of the total variation, whereas for the VRN gene, the sampling and extraction step showed an even distribution of the error compared to the other processing steps (~33% per step). This finding supports the notion that the spread in measured Cq values can have different origins, depending on the gene, even when the same subjects and tissues are analyzed. Human and animal research literature provides a substantial amount of evidence that observed expression differences can have different origins. Biological variance has been reported in human blood, lung, placenta, and retina, just to name a few (Cheung et al., 2003; Chowers et al., 2003; Whitney et al., 2003; Sood et al., 2006) and different variations of gene expression between genes expressed in the same tissue have been found in mouse liver and heifer blood (Pritchard et al., 2001; Tichopad et al., 2009; Vedell et al., 2011; Corton et al., 2012). It is commonly accepted that some genes are more tightly controlled (e.g., reference genes) while other genes (not necessarily responding to any study factor) may vary much more relative to their means. However, the nature of the variation in gene expression in specific tissues is an unexplored issue in plant biology. Here, we demonstrate that different levels of inherent variability exist in each step of the qPCR workflow, and therefore each step contributes differently to the background expression.

In summary, it seems that the error introduced by the sampling and extraction steps depends on the type of tissue (σ_Sampling_, 0.02–0.87 cycles; mean 0.44 cycles). With the exception described above for the gene F3′5′H, the sampling step shows greater variation than the RT or qPCR steps in stems; for fruits, RNA sampling contributes noise that is comparable to the other two steps; and for leaves, sampling shows higher reproducibility than the other steps (Figure 1).

### The common practice of qPCR replicates

**Figure 2:**
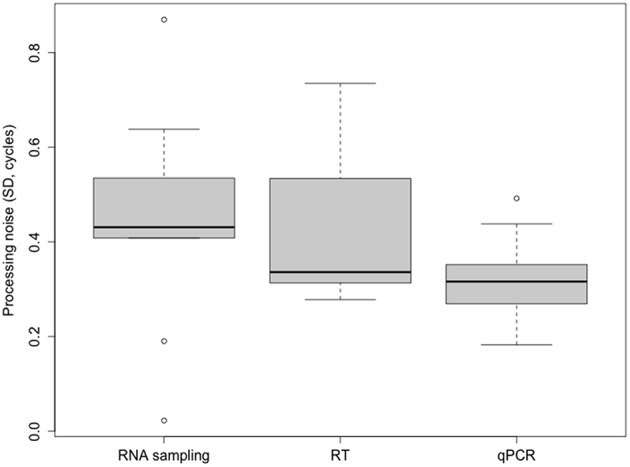
**Boxplot of the contribution to the overall noise by the sampling-processing steps**. Processing noise is expressed as the SD of measured Cq values. Variance is dominated by the sampling step, followed by the RT step. qPCR is the step with the highest reproducibility.

Unlike the error introduced by the sampling and RT steps that depends on the type of tissue, the extent of variation contributed by the qPCR step seems to be consistent for samples of all types, not being the main source of variation in any analyzed tissue. We find, for the last step of the workflow, σ_qPCR_ values of 0.18–0.49 cycles, with a mean of 0.32 cycles. The qPCR step showed the highest reproducibility in eleven out of the 18 combinations assessed, involving the three genes measured, the three tissues analyzed and the two individual plants studied. The noise introduced by the sampling or the RT (σ_RT_, 0.28–0.73 cycles; mean 0.43 cycles), on the other hand, suggests that the reproducibility of these steps is ~1.5 times less than that of the qPCR. The mean variance contribution from the qPCR step was 25.5%, while sampling and RT showed similar imprecision with 36.8 and 37.8% of the mean variance, respectively. Therefore, the qPCR is the step that consistently contributes the least to the combined noise (Figure 2).

The total confounding variance or SD σ_Cq_ can be reduced by performing technical replicates and by use of mean values in subsequent analysis to average-out processing noise (Tichopad et al., 2009). On the basis of the variance contributions that we have estimated for the three tissues from blueberry, we are able to better evaluate the importance of qPCR replicates. Performing a qPCR in duplicate reduces its noise contribution from 0.32 to 0.23 (Equation 3). This reduction is not a substantial improvement compared with the total observed processing noise. Also, the other sample-processing steps appear to contribute more to the total noise than the qPCR step, thus by using replicates at these other steps, the reproducibility of the measurement (that is, decrease in the total variance) will be more greatly improved.

### Experiment optimization

In terms of optimization of the experimental design, it is the objective to minimize the total expected variation, which is defined in Equation (4) as $\sigma_{\text{Cqg}}^{2}$. By varying the *n* replicates at each level, the $\sigma_{\text{Cqg}}^{2}$ changes. In our blueberry case study, the decision of where to place replicates depends on the tissue that is under analysis. For example, for *VRN*, the 1 × 2 × 4 × 2 experimental plan in leaves produces the same variance of the mean Cq as the 1 × 5 × 1 × 2 experimental plan would in stems (σ_Cqg_ = 0.33 cycles). The recommendation that can be extracted from our blueberry data is that increasing the number of RT replicates is superior to other types of replicates when working with leaves, while adding RNA extraction replicates is most beneficial when working with stems or fruits to reduce the total variance. Table 2 shows different examples of sampling plans for quantifying the *VRN* expression level in stem tissues. From these plans with 4, 8, and 12 total replicates, two conclusions can be drawn. First, for a given number of total replicates, the optimal design is not the one with the highest qPCR replicates, but the one that incorporates more upstream replicates. The second conclusion is that it is possible to have a lower total variance in an experimental design that uses a lower number of replicates than in some experimental designs that use a higher number of replicates, provided the replicates are performed in an optimal combination.

**Table 2:** **Optimization of a sampling plan for the *VRN* gene in blueberry stems**.

**Total replicates**	**Subject**	**Sampling**	**RT**	**qPCR**	**Total Var**.
12	2	1	2	3	0.61
12	2	1	3	2	0.56
12	2	2	3	1	0.30
8	2	1	2	2	0.63
8	2	2	1	2	0.41
8	2	2	2	1	0.34
4	2	1	1	2	0.82
4	2	1	2	1	0.67
4	2	2	1	1	0.45

*For any given number of replicates, a design that incorporates upstream replicates minimizes the expected total variation*.

The ultimate question is “what is the best experimental design that we can perform?” The answer is the one in which $\sigma_{\text{Cqg}}^{2}$ is minimized. Here, the total number of replicates and how they are combined come into play. One might intuitively think the greater the number of replicates the better. However, the decision of how many replicates are used in the actual experiment is a balance between accuracy and practicality related to the specific budget and time constraints. Therefore, even though each experimental step contributes to the total variance, it is not realistic to perform an “unlimited” number of replicates in each experimental step for the sake of variance reduction. The costs associated with the different experimental steps can be used as an external constraint to help find the optimal experimental design.

With this goal, we have created a script in R that considers the number of biological conditions, the variation from the different experimental steps extracted from the pilot experiment and the budget limitations (https://github.com/jdieramon/BlueberryProject). Knowing these variables as input data, the script estimates the optimal variance of the mean Cq that is expected under those assumptions and determines the optimal sampling plan for that value. For example, for genes exhibiting σ_qPCR_ = 0.3, σ_RT_ = 0.31, and σ_Sampling_ = 0.42, we might want to measure their expression levels in fruits at three different stages of development (green, pink and ripe stage). Assuming a cost of 1 unit for the qPCR, 3 units for the RT, 10 units for the sampling/extraction and 50 units for each plant, with a total budget of 1000 units, the best we can do is to analyze fruits from 3 plants per stage of development, sample each plant 4 times, perform 1 RT per RNA and 2 qPCR per cDNA. In this study, the total variation within each group is expected to be practically null (σ_Cqg_ = 0.07 cycles). If we used for the same study a single sample collected from a single plant (using 3 plants per developmental stage), performed 1 RT per sample and run qPCR in triplicates, the expected variation among plants is estimated to be σ_Cq_ = 0.28 cycles. The optimal plan uses 2.67 times more replicates than the second design and costs 1.67 times more monetary units but it reduces the variation 4-fold while keeping the final cost within the assigned budget.

## Concluding remarks

Since its introduction about 20 years ago, RT-qPCR has become the most common technique for RNA expression measurements. Despite the introduction of next-generation sequencing (NGS) for gene expression analysis in plant genomics (Jain, 2012; Die and Rowland, 2013), qPCR remains an essential technique for confirmation of NGS findings. One decade ago, increased awareness of problems associated with producing high-quality and reliable data from RT-qPCR came to the forefront (Bustin, 2002). It is not that qPCR is intrinsically inaccurate, it is in fact quite a stable reaction, but rather it is the lack of systematic procedures and performance of such procedures that can compromise results and conclusions (Derveaux et al., 2010). In the RT-qPCR workflow, samples must pass through a number of preparative steps prior to the qPCR assay itself, and each one can introduce variability. Reliable data can be produced only when the experimental variance is minimized, so the sources of noise need to be identified and optimized for each step (Reiter et al., 2011). In RT-qPCR, replication of the upstream processes are often disregarded in favor of only the qPCR reaction. Since error is introduced at each step of the process, however, these steps deserve closer attention. In this case study using blueberry, we aimed to identify the sources of the highest technical noise in order to ultimately better design experiments. For the assessment of variance, we used multiple experimental replicates at each step. In general, we found that the qPCR step contributed the least to the total variability, and depending on the tissue used, the sampling step or the RT step contributed the most to the total variation. Therefore, adding more replicates at one or both of these two earlier stages should be more beneficial in terms of minimizing the total experimental variance.

## Author contributions

JD participated in the design of the study, performed qPCR data analysis, made substantial contributions to interpretation of data and drafted the manuscript. BR conceived of the study, participated in its design and helped to draft the manuscript. FF performed the statistical analysis and interpretation of data. LR contributed reagents/materials/analysis tools, made substantial contributions to interpretation of data and drafted the manuscript. All authors read and approved the final manuscript.

### Conflict of interest statement

The authors declare that the research was conducted in the absence of any commercial or financial relationships that could be construed as a potential conflict of interest.

## Abbreviations

RT: reverse transcription
RTase: reverse transcriptase
qPCR: quantitative real-time PCR
RT-qPCR: reverse transcription–qPCR.
