# Supplementary material for: Design and Sampling Plan Optimization for RT-qPCR Experiments in Plants: A Case Study in Blueberry
Source: Front Plant Sci. 2016 Mar 7;7:271. doi: 10.3389/fpls.2016.00271 (PMC4779984; doi:10.3389/fpls.2016.00271)
Supplement: Supplementary file 1 [file Supplementary_Material.PDF]

Supplementary Material

Design and sampling plan optimization for RT-qPCR experiments in plants: a case study in blueberry

Jose V. Die1*, Belen Roman, Fernando Flores, Lisa J. Rowland

*** Correspondence:** Corresponding Author: Jose.Die@ars.usda.gov

1 Supplementary Figures and Tables

1.1 Supplementary Figures

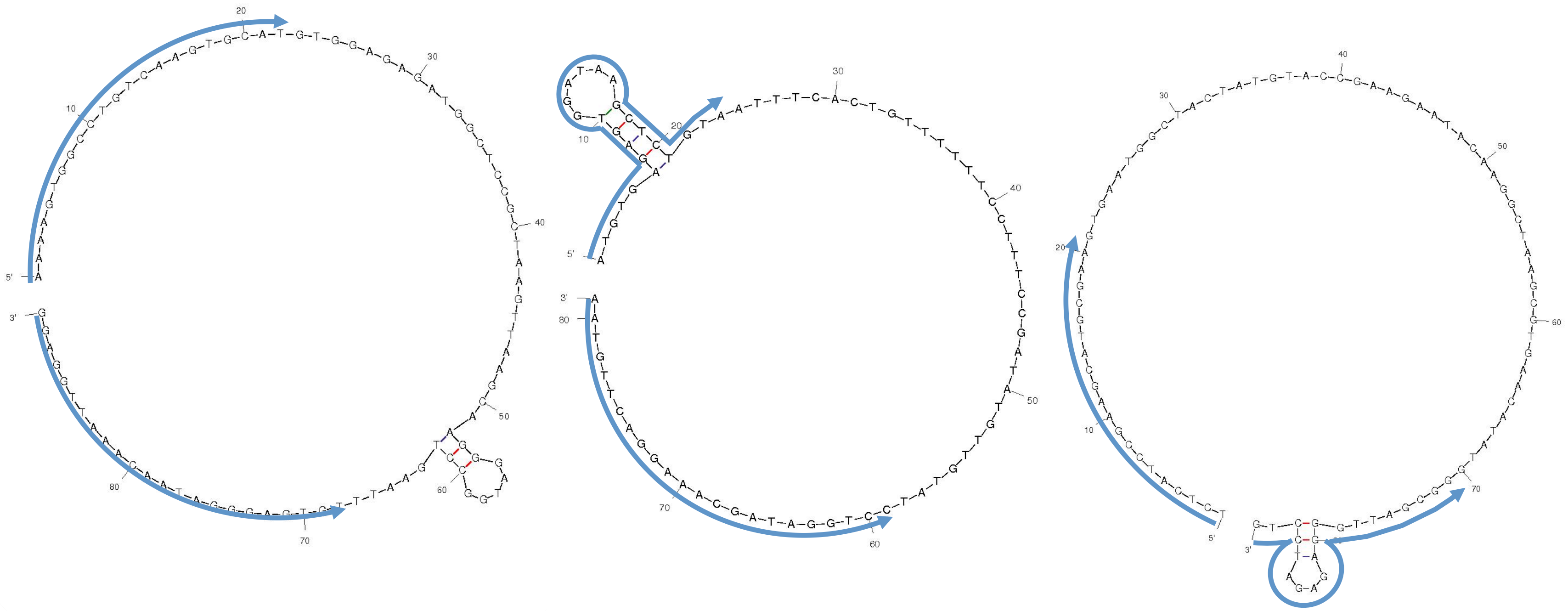

Supplementary Figure 1. Secondary structures of the amplicons for the 3 primer sets used in this study. Thermodynamic stability (ΔG in kcal/mole) is presented in the figures. Primers are indicated by blue arrows. No secondary structures are present where primer anneals and efficient annealing is not hampered in the *VRN* amplicon. The secondary structures at the primer annealing site for *KAT* forward primer and *F3'5'H* reverse primer have positive ΔG value and Tm <60ºC and hence do not influence the amplification efficiency.

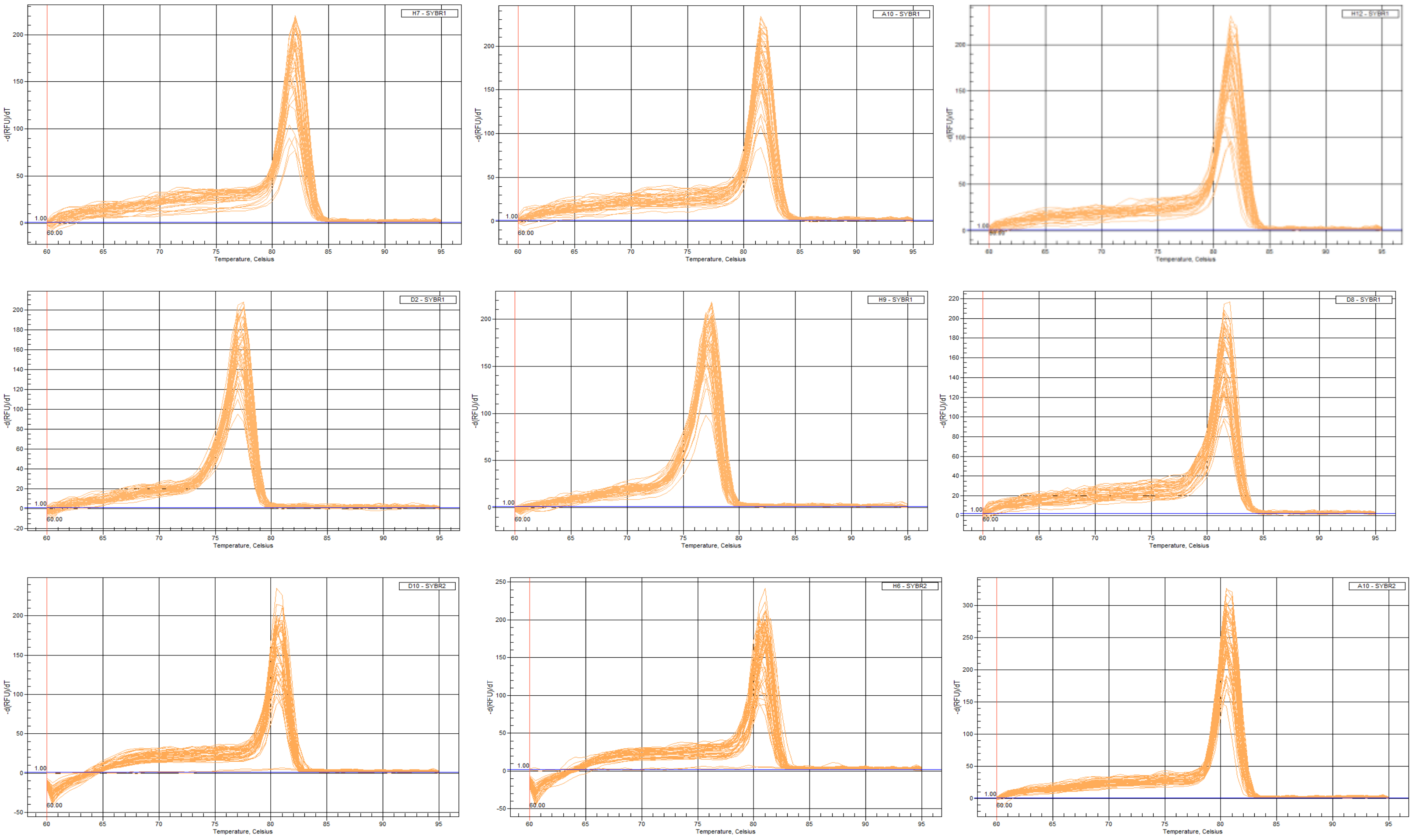

Supplementary Figure 2. Melting curves for representative samples. **A.** *VRN* gene in leaves, stems and fruits (Premiere). **B.** *KAT* gene in leaves, stems and fruits (Tifblue). **C.** *F3'5'H* gene in leaves, stems and fruits. (Premiere).

1.2 Supplementary Tables

Supplementary Table 1. Description of sequences used in this study.

Symbol	Gene Id.	Description	Identity	*E*-value	Species	GenBank Acc.
VRN	contig03351	B3 domain-containing TF *VRN*1-like	59%	7e-53	*Vitis vinifera*	XP_002281517
KAT	contig02401	3-ketoacyl-CoA thiolase	90%	4e-58	*Arachis diogoi*	ACA58356
F3'5'H	JK666610	flavonoid 3',5'-hydroxylase	85%	0.0	*Rhododendron x pulchrum*	BAF96946
	JK664585					
	JK666329					
	JK664236					

Blueberry 454 ESTs *VRN* and *KAT* sequences were obtained from http://bioinformatics.towson.edu/BBGD454/ (contig03351 in "all samples" library; contig02401 in "MID4" library). Four partial EST sequences obtained from blueberry fruit libraries were retrieved from GenBank and aligned to construct a full-length sequence that matched with a *F3'5'H* gene from *Rhododendron*.

Supplementary Table 2. Description of primer sequences used in this study.

Symbol	Primer Sequence (5'-3')	PCR product size	PCR Efficiency		
				Prem.	Tif.
VRN	F-AAAAGTGGCCTGTCAAGTGCAT<br>R-CCTCCAATTTGTTATCCCTCACA	90 bp	Leaves	0.94	0.94
			Stems	0.95	0.91
			Fruits	0.95	0.84
				Prem.	Tif.
ATK	F- ATGTGAGAGTGGATAAGCTCTGT<br>R- TTACAAGTCCTTTGCTATCCAGG	81 bp	Leaves	0.89	0.88
			Stems	0.90	0.87
			Fruits	0.85	0.86
				Prem.	Tif.
F3'5'H	F- TCTCATCCGAAGCATGCGAA<br>R- CAGGATCTCTCCCAATCGCC	90 bp	Leaves	0.88	0.91
			Stems	0.90	0.93
			Fruits	1.06	1.03

PCR E mean calculated according to the equation $(1+E) = 10^{slope}$ using LinRegPCR (Ramakers et al. 2003). Data represent mean values ($44 \leq n \geq 48$).
